# Relieving efforts in palm‐tree tissue sampling for population genetics analyses

**DOI:** 10.1002/ece3.7624

**Published:** 2021-05-11

**Authors:** Sebastian A. Espinoza‐Ulloa

**Affiliations:** ^1^ Facultad de Medicina Pontificia Universidad Católica del Ecuador Quito Pichincha Ecuador; ^2^ Department of Biology University of Saskatchewan Saskatoon SK Canada

**Keywords:** DNA methods, palm‐tree, population genetics, root tissue, sampling methods

## Abstract

1. The young leaves are the main source of nucleic acids for population genetic studies in palm‐trees; however, the access to this tissue may be limited by specific features of each species. Using root tissues as an alternative source of nucleic acids could facilitate the sampling in large populations.
2. This study tests root tissue viability as an alternative nucleic acid source (root versus. leaf) and explores different protocols (tissue storage and DNA extraction methods) to obtain high‐quality DNA samples.
3. The results showed no significant differences in DNA concentration (603.7 vs. 599.1 ng/μl) and quality ratios (A260/280:2.1 vs. 1.9, and A260/230:2.1 vs. 2.0) for the comparisons of tissue source (leaf vs. root) and DNA extraction method (manual vs. kit). For tissue storage method, DNA concentration was significantly higher for root tissues stored in 70% and 90% alcohol solutions (692.8 and 822.6 ng/μl, respectively) versus those obtained from leaf tissue (603.7 ng/μl); however, for the quality parameters, no differences were found.
4. Results showed the effective potential of using root tissue as an alternative source for nucleic acids, which could facilitate population sampling of palm‐tree species for future studies, and this methodological alternative could be applied to other plant systems with similar sampling challenges.

​

## Graphical abstract

This study tests root tissue viability as an alternative nucleic acid source (root vs. leaf) and explores different protocols (tissue storage and DNA purification methods) to obtain high‐quality DNA samples. Results showed the effective potential of using root tissue as an alternative source, which could facilitate populations sampling of palm‐tree species.

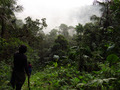

## 1 INTRODUCTION

For population genetics, the experimental design for plant tissue sampling can be affected mainly by individuals' density, the proximity between individuals, the topography, and the access to the tissue to be sampled. Tissues with high cell division rates are the best option in order to get a DNA sample of good concentration and quality, mainly due to the high amount of cells and high replication activity (Lucas et al., 2019; Tapia‐Tussell et al., 2005). Conventionally in plants, tissue samples with high concentrations of nucleic acids are obtained from meristematic tissue of new shoots or leaves (Edwards et al., 1991; Tai & Tanksley, 1990; Tapia‐Tussell et al., 2005). In palm‐trees, meristematic tissues, in general, are only found in the crown and roots (Arif et al., 2010; Broschat & Donselman, 1990). Traditionally, tissue sampling for DNA extraction is obtained from young leaves (found only in the crown). However, for most species, access to this tissue requires an extraordinary sampling effort due mainly to stem height, stem modifications (spines or prominent scars of old leaves), and/or even to high degrees of epiphytism in addition to habitat factors which also can further increase sampling efforts. Such increases in sampling effort often lead to modifications in the experimental design in terms of the number of individuals sampled or the time designated for it (Ihase et al., 2016; Lowman et al., 1993). Moreover, easier access to a meristematic tissue for sampling palm‐trees would be found in their roots, where it is expected to get similar DNA concentration and quality as is obtained from leaves (Broschat & Donselman, 1984; Jouannic et al., 2011). However, the tissue and cell organization between leaf and root are very different, and therefore, the treatments for obtaining optimal DNA for molecular analysis may differ. The tissue in the leaves is arranged in parallel cell layers forming a horizontal structure, which would allow rapid drying of the tissue and the preservation of all cellular structures using Silica Gel (method mostly used for the collection of tissue from leaves for molecular analysis; Arif et al., 2010; Tai & Tanksley, 1990; Tapia‐Tussell et al., 2005). While the root is made up of cell layers that form a succulent cylindrical structure, which under the same treatment used in leaves, the desiccation would be slower allowing the degradation of the tissue and would lead to a low yield in obtaining DNA (Bressan et al., 2014; Jouannic et al., 2011; Tapia‐Tussell et al., 2005).

Taking into account the problems that arise for several palm‐tree species, regarding the access to young leaves (or meristematic tissue associated with the crown) and their possible negative implications on the sampling effort for population genetics studies, it is thus necessary to explore alternatives that reduce this effort. For instance, in palm‐trees, root tissue would be theoretically an ideal candidate to obtain high concentrations of nucleic acids. However, previous trials have had not obtained satisfactory results using roots as source of DNA (Lucas et al., 2019). This may have been due to different treatments in sample collection, storage, and\xA0processing were not tested. The root apex is mainly formed by meristematic tissue that is in constant formation and replacement (Broschat & Donselman, 1990). Therefore, it would be expected the terminal region of the root contains high concentrations of nucleic acids mainly from nonspecialized cells (Broschat & Donselman, 1984, 1990; Jouannic et al., 2011). This study aims to test the use of root tissues as an alternative source of nucleic acids. It thus also seeks to establish the best tissue storage and DNA extraction methodologies.

## 2 MATERIALS AND METHODS

Due to the fact that the present study will have applications in future studies of several Andean palm‐tree species (Arecaceae), four different species of easy access were selected in the surroundings of Quito city (Ecuador) in order to obtain a proxy of possible variations between species. The species were as follows: (a) *Phoenix* *dactylifera*, (b) *Ceroxylon echinulatum*, (c) *Ceroxylon ventricosum,* and (d) *Prestoea acuminata*. These species are easily found in the Ecuadorian Andean urban and rural landscapes (between 1,800 and 2,500 m.a.s.l.). All these species are conspicuous stem palm‐trees that commonly exceed 10 m in height, and their roots are placed underground.

**FIGURE 1:**
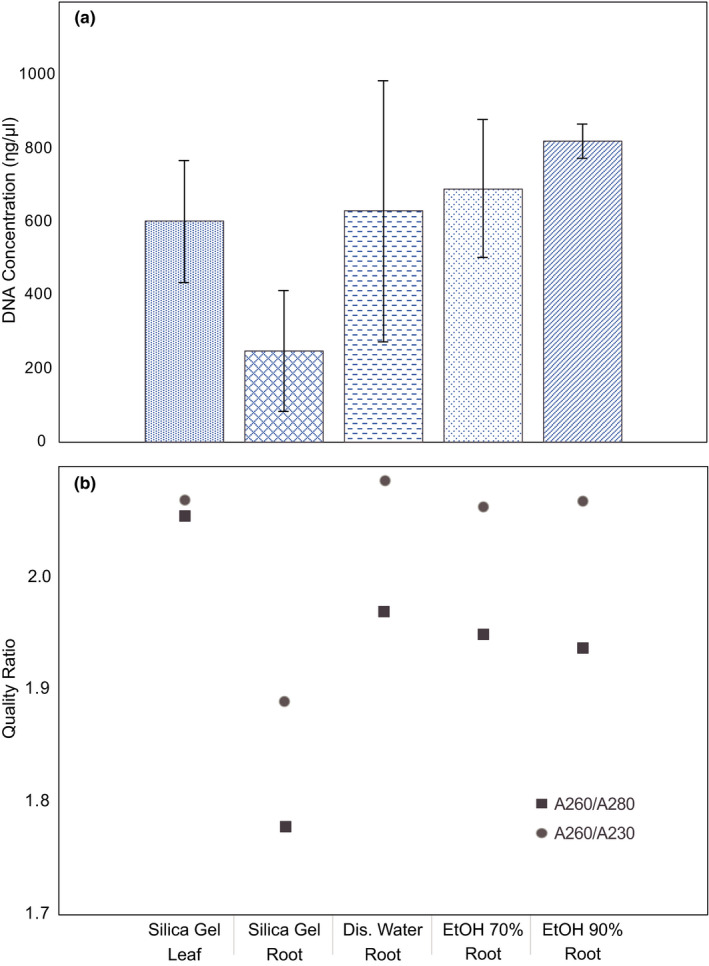
(a) DNA concentration (ng/μl) means and 95% confidence intervals for each tissue treatment. (b) Quality ratios, A260/280 (squares) and A260/230 (circles), for each tissue treatment. Tissue treatments are set as follows: (1) leaf–Silica Gel (*n* = 48), (2) root–Silica Gel (*n* = 48), (3) root–distillated water (*n* = 48), (4) root–EtOH 70% (*n* = 48), and (5) root–EtOH 90% (*n* = 48)

For sampling, two individuals were selected per species. The tissue collection for each individual was carried out (a) climbing along the stem to obtain young leaf tissue and (b) using a shovel to dig about 30 cm in the stem base to obtain root tissue (Figure S1). Leaf tissue was stored directly in a single bag with Silica Gel, following the most commonly used protocol for leaf tissue sampling (control sample). For root tissue sampling, eight roots per individual were pruned at 5 cm from the tip (Figure 1), and two pruned roots were placed into each tissue storage treatment. Four storage treatments for root tissues (T1 to T4) were used as follows: (T1) Silica Gel dehydration (rationale: standardized method for plant tissue sampling), (T2) distilled water (rationale: maintain hydrated and alive the tissue as is possible), (T3) 70% ethanol solution (EtOH 70%; rationale: dehydration by alcohol in a liquid solution), and (T4) 90% ethanol solution (EtOH 90%; rationale: stronger dehydration by alcohol in a liquid solution). All samples were processed after 2 weeks of storage.

Each stored tissue underwent two DNA extraction methods: (a) DNA extraction manual method for plant tissues based on Doyle and Doyle (1987, with modifications) and (b) commercial method using PureLink® Plant Total DNA Purification Kit (Invitrogen™). Each extraction started with 100 mg of macerated tissue. The final DNA extraction solutions were evaluated using NanoDrop™ 1000 Spectrophotometer (Thermo Fisher Scientific). Three readings for each processed sample were taken.

DNA concentration and quality were compared between tissue sources, storage treatments, and DNA extraction methods. The concentration was measured as DNA nanograms contained in one microliter. The quality parameters are based on the A260/A280 and A260/A230 ratios, where A260 is the absorbance at 260 nm that would be marking the presence of aromatic bases (nucleotides/DNA/RNA), A280 is the absorbance at 280 nm that would recognize contaminants such as proteins and phenolic compounds, and A230 would identify residual contaminants of many organic compounds such as phenol, TRIzol, salts, among others. In this way, the A260/A280 ratio would be linked to factors related to the nature and processing of the tissue, while the A260/A230 ratio would be linked to the reagents and residues produced by the extraction protocol used (Matlock, 2015).

Finally, using all DNA extractions obtained, a positive–negative PCR test was performed. The PCR test was performed with two microsatellite loci developed for *Oenocarpus bataua* (*Ob01* and *OB11*; Montufar et al., 2007) that had previously been tested and had positive results for all species used in this study. The amplification parameters for the used loci were the same described in their source paper.

For the statistical analyses, a multifactor ANOVA (general linear model) was performed to observe the statistical differences between the analyzed factors. The factors analyzed were as follows: (a) species, (b) source (leaf vs. root), (c) storage treatment, and (d) extraction method. Additionally, a Tukey post hoc test was performed to compare the results obtained by each storage treatment versus the results obtained for the leaf, in order to determine the best storage treatment and how much it differs from the result of a standard protocol.

## 3 RESULTS

For all the samples used, positive results were obtained for DNA extraction. The results for the concentration showed relatively high values for all samples, with more than 95% of the samples above 100 ng/µl. The leaf tissue mean DNA concentration was 603.71 ng/µl, while for root tissue was 599.12 ng/µl. For quality, the average for leaf was 2.06 for A260/280 ratio and 2.08 for A260/230 ratio, while for root was 1.91 and 2.03, respectively (Table 1).

**TABLE 1:** Descriptive statistics for concentration and quality of DNA obtained

Source	Treatment	*n*	Concentration (ng/μl)		Ratio A260/A280		Ratio A260/A230	
			Mean	Std. dev.	Mean	Std. dev.	Mean	Std. dev.
Leaf	Silica gel	48	603.714	164.46808	2.0556	0.43211	2.0698	0.34951
Root	EtOH 70%	48	692.8408	188.07232	1.9502	0.40718	2.0631	0.35794
	EtOH 90%	48	822.571	298.89048	1.9373	0.35867	2.0685	0.25334
	Silica gel	48	249.7406	165.67411	1.7781	0.64947	1.8894	0.56363
	Distillated water	48	631.3379	355.81995	1.9696	0.42652	2.0869	0.3602
	Root overall	192	599.1226	338.0658	1.9088	0.47631	2.027	0.40478

Regarding the storage treatments, the leaf DNA concentration (603.71 ng/µl) was lower than all liquid storage treatments for roots: EtOH 90% (822.57 ng/µl), EtOH 70% (692.84 ng/µl), and distilled water (631.34 ng/µl; Table 1; Figure S2). On the other hand, for quality, the highest values were for distilled water storage treatment with the root, where the A260/280 ratio was 1.97 and the A260/230 ratio was 2.09, while in leaf they were 2.06 and 2.07, respectively (Table 1; Figure 1).

The statistical comparison by ANOVA found no significant differences for DNA concentration between leaf and root tissues (Table 2). However, the analysis showed significant differences for DNA concentration between species and storage treatments, also the comparison between extraction methods was close to significance limit (*p* = .050; Table 2). Regarding the A260/280 ratio, there was a significant difference between species, while for tissues the probability was near to the significance limit (*p* = .053). Finally, for the A260/230 ratio, no significant differences were found for all comparisons carried out (Table 2).

**TABLE 2:** ANOVA *p‐*values summary for each comparison

*Comparison*	Concentration (ng/μl)	Ratio A260/A280	Ratio A260/A230
Palm species	0.000**	0.010*	0.297
Source (leaf versus root)	0.927	0.053	0.502
Storage treatments	0.000**	0.065	0.078
Extraction method	0.050	0.468	0.132
Leaf versus root (EtOH70)^a^	0.396	0.802	1
Leaf versus root (EtOH90)^a^	0.000**	0.725	1

^a^Obtained from Tukey post hoc test.

* Values are significant at *p* < .05

** Values are significant at *p* < .01.

As a final result, although no statistical tests were performed, the PCR test for all samples obtained was positive, showing a unique expected amplicon in the agarose gel (2%) for each DNA extraction and for each locus (e.g., Figure S2). Which could prove the DNA solutions obtained did not contain important reaction inhibitors.

## 4 DISCUSSION

This study has shown obtaining DNA from root tissue is possible and it could be a plausible alternative to the leaf tissue to reduce sampling efforts. Regarding the source tissue, no differences for DNA concentrations were found between the overall root results with leaf tissue results. However, for quality, the A260/280 ratio is close to the limit of significance (*p* = .053). This almost significant difference may be explained by the higher amount of contaminants (mainly proteins) linked to the root tissue. In most monocotyledons, the roots are constantly growing, and to maintain their structure underground, the tissues need to be constituted by higher concentrations of lignin (and other related proteins) compared with the leaves (Abiven et al., 2011; Hans‐Walter & Piechulla, 2011; Merewitz et al., 2011). For this reason, it would be expected that the DNA extraction quality from the root is somewhat lower than that was obtained from the leaf tissue. However, the quality could be improved with standardized storage condition and adjusting the extraction protocol.

Regarding the root storage treatments, the general comparison showed significant differences for DNA concentration, while for both quality ratios, there were no differences. This would be explained by the degree of stabilization and fixation of nucleic acids that would result from the different treatments used. Within treatments, the most satisfactory outcomes for DNA concentration were yielded by both ethanol solutions (70% and 90%). However, a specific comparison between leaf tissue and root tissue stored in ethanol 90% showed a significant higher DNA concentration recovered from the root. Ethanol is a tissue fixative, since it dehydrates the tissue violently (exchanging the water from the sample) maintaining the cellular structure and precipitating the nucleic acids; in consequence, higher concentration of ethanol allows to recover more DNA (Bressan et al., 2014). On the other hand, the least effective result was that obtained with Silica Gel. Silica Gel salt works by dehydrating the tissue to fix the cells and all their components, and it has been a great tool for the fixation of leaf tissues in plants. However, its low effectiveness in root DNA extraction could be explained by the speed of tissue dehydration. Leaf tissue is arranged in layers forming a single almost regular structure, while the root tissue sample is an irregular and cylindrical structure. Therefore, the root sample will take more time for dehydration, which would allow the breakdown of nucleic acids (Arif et al., 2010; Bressan et al., 2014; Jouannic et al., 2011; Tai & Tanksley, 1990).

Finally, the comparison between species demonstrated significant differences for concentration, as well as for quality (A260/280), which would be explained by the intrinsic characteristics of each species. Tissues of each species will differ in their shape, composition, and cellular structure; therefore, it is understandable that a specific quantity and quality of DNA are recovered from each species according to the specific nature of their tissues (Abiven et al., 2011; Tapia‐Tussell et al., 2005).

This short experiment proved that it is possible to obtain a “good” DNA sample from palm‐tree root tissue, which was additionally supported by the PCR tests performed. Positive PCR tests showed that the resulting DNA extractions are free of contaminants or reaction inhibitors and suggest that the constitution of the genetic material was not significantly degraded (Edwards et al., 1991; Lucas et al., 2019). In conclusion, the DNA extraction from root tissue in palm‐trees is strongly feasible and this fact could facilitate palm‐tree population sampling for large‐scale studies by reducing the sampling efforts. Additionally, the storage treatment experiments provide a basis for development of an effective, standardized protocol to obtain best DNA possible for further processing (even for genomic analyses) despite species or DNA extraction method.

## CONFLICT OF INTEREST

The author declares that there is no conflict of interest.

## AUTHOR CONTRIBUTIONS

**Sebastian A. Espinoza‐Ulloa:** Conceptualization (lead); data curation (lead); formal analysis (lead); investigation (lead); methodology (lead); validation (lead); visualization (lead); writing‐original draft (lead); writing‐review & editing (lead).

### OPEN RESEARCH BADGES

This article has earned an Open Data Badge for making publicly available the digitally‐shareable data necessary to reproduce the reported results. The data is available at https://doi.org/10.22541/au.160595652.27877911/v1.

## Supporting information

Fig S1‐S2

## Data Availability

The dataset as well as the detailed statistics output is available at Dryad (https://doi.org/10.5061/dryad.ht76hdrfj).
