# Supplementary material for: Relieving efforts in palm‐tree tissue sampling for population genetics analyses
Source: Ecol Evol. 2021 May 11;11(12):7946–50. doi: 10.1002/ece3.7624 (PMC8216967; doi:10.1002/ece3.7624)
Supplement: Supplementary file 1 — Fig S1‐S2 [file ECE3-11-7946-s001.docx]

**Relieving efforts in palm-tree tissue sampling for population genetics analyses.**

**Sebastian A. Espinoza-Ulloa**

**Supplementary material**


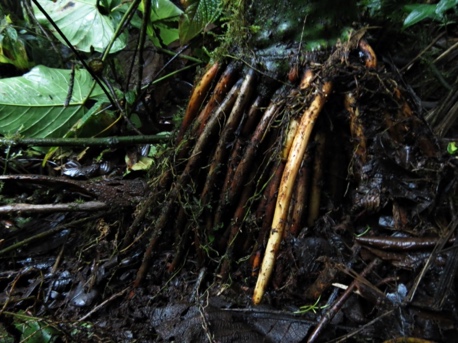


Supplementary figure 1. A) For root sampling, the soil in the base of the palm-tree stem was removed digging about 30 cm to expose the roots. B) The tissue sampling consisted of pruning the final 5 cm of the root and storing it under the different treatments.


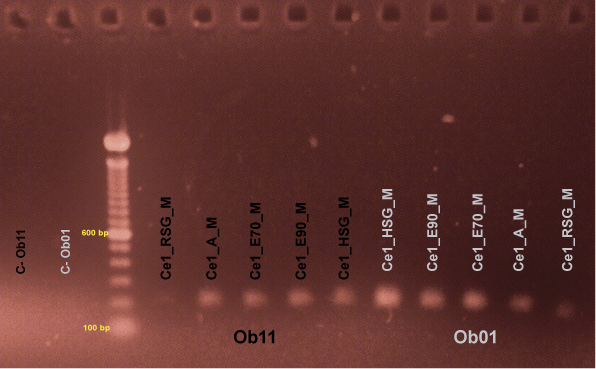


Supplementary figure 2. PCR test with two microsatellites (*Ob01* and *OB11*; Montufar *et al*., 2007) for one individual (Ce1, *Ceroxylon echinulatum*) for leaf extraction and for each root storage treatment. All samples were extracted with manual method (Doyle & Doyle, 1987). For *Ob11* (black), the samples arranged from left to right are: 1) root in silica gel, 2) root in distilled water, 3) root in ethanol 70%, 4) root in ethanol 90%, and 5) leaf in silica gel. For *Ob01* (white), the samples arranged from left to right are: 1) leaf in silica gel, 2) root in ethanol 90%, 3) root in ethanol 70%, 4) root in distilled water, and 5) root in silica gel. At the ladder left side are the negative controls for each microsatellite.
